# A Combination of Surgical Techniques to Repair a Giant Traumatic Macular Hole

**DOI:** 10.1155/2018/7595873

**Published:** 2018-12-09

**Authors:** Soon Wai Ch'ng, Ibrahim Elaraoud, David Karl, Dimitrios Kalogeropoulos, Rynn Lee, Elisa Carreras

**Affiliations:** Birmingham Midlands Eye Centre, Sandwell and West Birmingham Hospitals NHS Trust, Dudley Road, Birmingham B18 7QH, UK

## Abstract

A 38-year-old man with a traumatic full-thickness macular hole (FTMH) presented to our eye casualty department with a sudden deterioration of his right eye vision to hand movements over the past one week. The suspected traumatic FTMH was present since he was 13 years old from a direct impact of a golf ball in his right eye and his best-corrected visual acuity (BCVA) has always remained at 1/60 Snellen vision. On examination, he had a very large FTMH measuring 1635 *µm* with central foveal retinal detachment. Pars plana vitrectomy combined with large inverted internal limiting membrane (ILM) peel flap, 5000 Cs silicone oil tamponade, and autologous platelets implantation was performed. Follow-up visits revealed that the FTMH was closed under silicone oil. The silicone oil was removed six months after the surgery and the FTMH remained close with the retina remaining attached. His BCVA was restored to his previous baseline level of 1/60 Snellen vision. With the advent of multiple techniques to repair FTMH such as the ILM flaps, we have combined this technique with older proven techniques such as silicone oil tamponade and autologous platelets implantation to close the giant traumatic FTMH. This case study demonstrates that combining techniques can help close a FMTH that is otherwise deemed impossible in the past.

## 1. Introduction

Traumatic FTMH has been reported to be in the incidence of 1-9% and more common in the younger male population [1–4]. In most cases of traumatic FTMH, surgical repair is often delayed due to the possibility of spontaneous closure [5–9]. In our case, the FTMH was present for more than 10 years and as there was an associated foveal RD and deterioration of vision, we have decided to proceed with surgical repair of the FTMH to prevent the loss of his peripheral visual field.

## 2. Case Study

**Figure 1:**
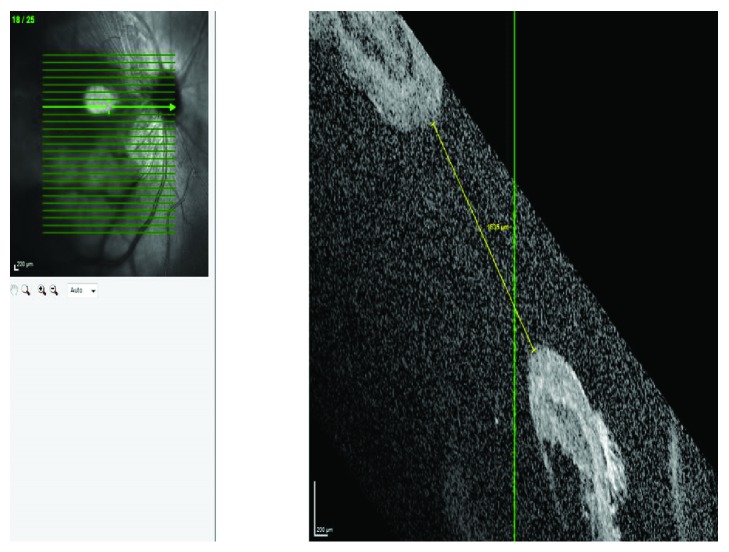
Optical coherence tomography (OCT) demonstrating the size of the large traumatic FTMH. There is an associated foveal retinal detachment around the traumatic macular hole.

**Figure 2:**
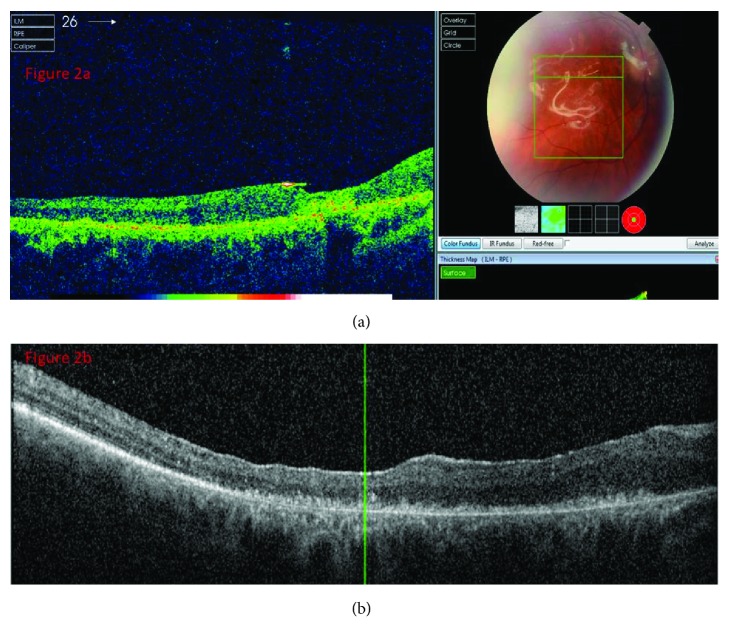
OCT demonstrating the closure of the FTMH before (a) and after (b) silicone oil removal.

A 38-year-old man with traumatic macular hole presented to the emergency eye department with a sudden painless deterioration of his right vision to hand movements (HM) and loss of peripheral vision for the past four days. He denied any recent trauma but past ophthalmic history revealed a suspected traumatic full-thickness macular hole (FTMH) which was present since he was 13 years old from a direct impact of a golf ball. The best-corrected visual acuity (BCVA) in the right eye had always remained at 1/60 Snellen vision after the accident. There was no other significant past medical history. On examination, his BCVA in his right eye was HM vision and left eye is 6/6. Anterior segment and intraocular pressure were normal in both eyes. Crystalline lenses were clear in both eyes. Dilated fundal examination in the right eye revealed a large FTMH measuring 1635 *µ*m with central foveal retinal detachment extending to the arcades (Figure 1). There were no peripheral retinal tears found. A partial posterior vitreous detachment (PVD) was present. There was also a Bergmeister's papilla. The left retina was normal. Following informed consent with the patient regarding the guarded risk of visual prognosis for the surgery, a pars plana vitrectomy (PPV) approach to repair the FTMH was performed. Autologous platelets were collected on the day of the surgery through a process of centrifugation of platelet rich plasma from whole blood. The surgery was performed under general anaesthesia. A standard 23-gauge three-port sclerotomy was performed. Core vitrectomy was performed followed by the induction of a PVD. A PVD that extended as far as possible to the vitreous base was created, followed by vitreous shaving with scleral indentation. The presence of PVD was confirmed with the use of intravitreal triamcinolone acetonide. The internal limiting membrane was stained with dual membrane blue dye. A large internal limiting membrane (ILM) inverted flap of two disc diameters was created to cover the FTMH. Following that, fluid-air exchange was performed and an internal tamponade of 5000 Cs silicone oil was inserted. Four droplets of autologous platelets were injected into close to the FTMH before the surgery was completed. All sclerotomy sites were sutured. At the end of the procedure, subconjunctival cefuroxime and dexamethasone were injected. Postoperative combined antibiotic-steroid drops (dexamethasone 1mg/ml, neomycin sulphate 3500 IU/ml, and polymyxin B sulphate 6000 IU/ml) were used 4 times a day for four weeks and cyclopentolate 1% drops was used 2 times a day for one week. The patient was instructed to position his face down for one week. One month after the surgery, the FTMH had closed under silicone oil (Figure 2(a)) but a cataract had developed. After six months, the silicone oil was subsequently removed together with a phacoemulsification and intraocular lens implantation. The FTMH remained closed with the retina attached (Figure 2(b)). His BCVA improved back to his baseline level of 1/60 Snellen vision.

## 3. Discussion

Since the advent of PPV to repair FTMH by Kelly and Wendell [10], closure rate of FTMH especially idiopathic ones has improved and reported to be in the range of 85 to 100% [11–14]. Closure rate of traumatic FTMH is reported to be about 85% with a single operation [15]. There are multiple surgical techniques that have been described to close idiopathic FTMH that remains open after the first surgery, traumatic FTMH, and Stage IV FTMH. The current surgical techniques include standard PPV to remove the posterior hyaloid, ILM peeling, and intraocular gas. Other additional steps include the use of silicone oil tamponade [16], autologous platelets implantation [17, 18] to the most recent techniques such as the inverted ILM flaps [19–21]. Traumatic FTMH from a blunt ocular trauma is formed from a contrecoup mechanism where a sudden decrease in the globe's anterior-posterior diameter with a compensatory equatorial expansion leads to horizontal forces and splitting of the retinal layers at the fovea. This causes an irregular configuration of the hole in a traumatic FTMH compared to an idiopathic FMTH [15]. As our patient had a giant FTMH, we have decided to proceed with the combination of multiple surgical techniques that have been described in the literature to maximise the chance of the hole closure. The techniques include the addition of a wide inverted ILM flap, silicone oil tamponade, and autologous platelets implantation to the standard surgical repair of a FTMH. Inverted ILM flaps are useful for large FTMH because it has been hypothesised that if a segment of the ILM is left attached to the FTMH, it will provoke gliosis inside the retina and surface of the ILM as well as providing a scaffold for tissue proliferation [20–22]. The indication of the use of autologous platelets was similar to the inverted ILM flap which was to further stimulate the glial cell proliferation in the hole to aid closure and this was first described to be successful by Chow et al. where 94% of their 16 eyes with traumatic FTMH achieved hole closure [23]. Although gas would have provided a better surface tension and visual outcome in most patients, we felt that our patient would have difficulty adopting his strict face down posture to achieve hole closure. Therefore silicone oil tamponade was used instead. Furthermore, silicone oil use in traumatic FTMH has been shown to have a comparable closure rate in a retrospective study by Bor'i et al. of 90% compared to 94% in perfluoropropane gas [16]. In our case, the combination of long-term tamponade and the addition of two techniques to promote glial cell proliferation in the hole led to the successful closure of the FTMH and flatten the retina.

This case study demonstrated that combination of surgical techniques can help close a FMTH that would have been deemed impossible before in the past. However, these techniques ideally should be tailored to each individual case as it may not be indicated in all patients.
